# Influence of Alcohol on the Intestinal Immune System

**DOI:** 10.35946/arcr.v45.1.03

**Published:** 2025-03-14

**Authors:** Henriette Kreimeyer, Cristina Llorente, Bernd Schnabl

**Affiliations:** 1 Department of Medicine, University of California San Diego, La Jolla, California; 2 Department of Medicine, U.S. Department of Veterans Affairs San Diego Healthcare System, San Diego, California

**Keywords:** alcohol, alcoholic liver disease, immune system, intestine, dendritic cells, macrophages, T-lymphocytes, anti-microbial peptides

## Abstract

**PURPOSE:**

Alcohol misuse is associated with disruption of the microbial homeostasis (dysbiosis) and microbial overgrowth in the gut, gut barrier disruption, and translocation of microbes into the systemic circulation. It also induces changes in regulatory mechanisms of the gut, which is the largest peripheral immune organ. The gut-liver axis is important for health and disease, and alterations in the intestinal immune system contribute to alcohol-associated liver disease (ALD). Understanding these changes might help discover new targets for drugs and therapeutic approaches.

**SEARCH METHODS:**

A systematic literature search was conducted in PubMed, Medline, and Embase of manuscripts published between January 2000 and November 2023 using the terms (“alcohol” or “ethanol”) AND (“immune” or “immunol”) AND (“intestine,” “colon,” or “gut”). Eligible manuscripts included studies and reviews that discussed the effects of ethanol on immune cells in the intestine.

**SEARCH RESULTS:**

A total of 506 publications were found in the databases on November 20, 2023. After excluding duplicates and research not covering ALD (415 articles), 91 studies were reviewed. Also included were manuscripts covering specific immune cells in the context of ALD.

**DISCUSSION AND CONCLUSIONS:**

Balancing immune tolerance vs. initiating an immune response challenges the intestinal immune system. Alcohol induces disruption of the intestinal barrier, which is accompanied by a thicker mucus layer and reduced anti-microbial peptides. This leads to longer attachment of bacteria to epithelial cells and consequently greater translocation into the circulation. Bacterial translocation activates the immune system, reducing the activity of regulatory T cells and inducing T helper 17 response via a variety of pathways. The role of innate immune cells, especially Type 3 innate lymphoid cells, and of specific B- and T-cell subsets in ALD remains elusive. Gut dysbiosis, translocation of viable bacteria and bacterial products into the circulation, and changes in the intestinal barrier have been linked to immune deficiency and infections in patients with cirrhosis. Modifying the intestinal immune system could reduce intestinal inflammation and alcohol-induced liver injury. Understanding the underlying pathophysiology can help to detect new targets for drugs and design therapeutic strategies.

The intestine is the largest peripheral immune organ. The intestinal immune system is shaped by gut microbiota colonizing the intestine and distinguishes between commensal bacteria that colonize the gut in the absence of disease and pathogenic (disease-causing) bacteria. It can either defend the body against a specific pathogen or foreign substance (immune response) or tolerate the pathogen or foreign substance (immune tolerance).^1^ The intestinal immune system consists of specialized epithelial cells that form a physical barrier and immune cells located in the lamina propria (a thin layer of connective tissue beneath the epithelium) that act as the first line of defense against invading pathogens (Figure 1). The intestinal epithelial cells are linked by tight junctions that prevent the passage of large molecules and viable microbes from the intestinal lumen through the mucosa to the systemic circulation (translocation).^2^ Tight junctions are composed of several proteins, including occludin, zonula occludens^1^, and claudins.^3^

Chronic alcohol consumption is associated with disruption of the microbial homeostasis in the gut (i.e., intestinal dysbiosis), which in turn alters the intestinal immune system (Figure 2). Alcohol and its metabolites directly and indirectly disrupt tight junctions, compromising the integrity of the intestinal epithelial barrier. This results in a condition termed intestinal barrier dysfunction that is characterized by increased permeability and the translocation of microbes and microbial products from the gut lumen to the extraluminal space (i.e., microbial translocation). This disruption causes immune system disturbance and an increase in production of inflammatory cytokines, both locally and systemically, which in turn aggravate intestinal barrier dysfunction. Changes in the intestinal barrier also can result in decreased nutrient absorption.^4^, ^5^

The gut-liver axis encompasses bidirectional communication pathways between the gut and the liver via the portal vein and the biliary system. The portal vein carries most of the venous blood from the intestine directly to the liver, whereas bile produced by the liver is delivered to the small intestine through bile ducts. The portal vein transports alcohol to the liver, where it is metabolized into acetaldehyde and acetate.^4^–^6^ The special connection between the gut and the liver allows alcohol to harm the liver directly but also permits translocation of pathogens, microbial associated molecular patterns (MAMPs) such as lipopolysaccharides (LPS), and immune cells, leading to alcohol-associated liver disease (ALD).^6^ ALD describes a disease spectrum ranging from simple steatosis, steatohepatitis with fibrosis, and cirrhosis to alcohol-associated hepatitis, an acute chronic liver disease with high mortality.^7^ ALD has been linked to changes in the intestinal bacterial population (microbiome), disruption of the intestinal barrier, and microbial translocation.^8^, ^9^ Treatment with 5-aminosalicylic acid, which ameliorates inflammation in the intestine, also reduces liver damage, emphasizing the importance of gut inflammation and the intestinal immune system for the pathogenesis of ALD.^10^

This review provides a comprehensive overview of the cells comprising the intestinal barrier and the intestinal immune system and how they are affected by alcohol. Due to the close relationship between the gut and liver, this review focuses on recent discoveries regarding the involvement of the intestinal immune system in ALD.

## Search Methods

Three databases (PubMed, Medline, and Embase) were searched on November 22, 2023, using the keywords (“intestine,” “colon,” or “gut”) AND (“immune” or “immunol”) AND (“ethanol” or “alcohol”) in the title or abstract. Articles published in the last 20 years (January 2003 to November 2023) were included.

## Results of the Literature Search

The literature search yielded a total of 506 publications. After excluding articles not covering effects of alcohol on the gut or the liver (287 articles) and duplicates (128 articles), 91 papers remained and were included in the review. Eligible studies and reviews focused on the effects of alcohol on immune cells in the intestine.

The following sections briefly review the functions of various intestinal epithelial and immune cells, incorporating insights gathered from the literature search to provide context. Throughout the review, the terms “alcohol” and “alcohol-associated liver disease” are used for human studies and general conclusions, and “ethanol” and “ethanol-induced liver disease” apply to animal studies. Chronic ethanol feeding in rodents typically refers to either the Lieber-DeCarli diet (a liquid diet that provides up to about one-third of the calories from ethanol) or the National Institute on Alcohol Abuse and Alcoholism (NIAAA) model diet (a 10-day Lieber-DeCarli diet combined with a single binge of 5 g/kg body weight ethanol).

## Intestinal Barrier and Alcohol-Induced Alterations

**Figure 1:**
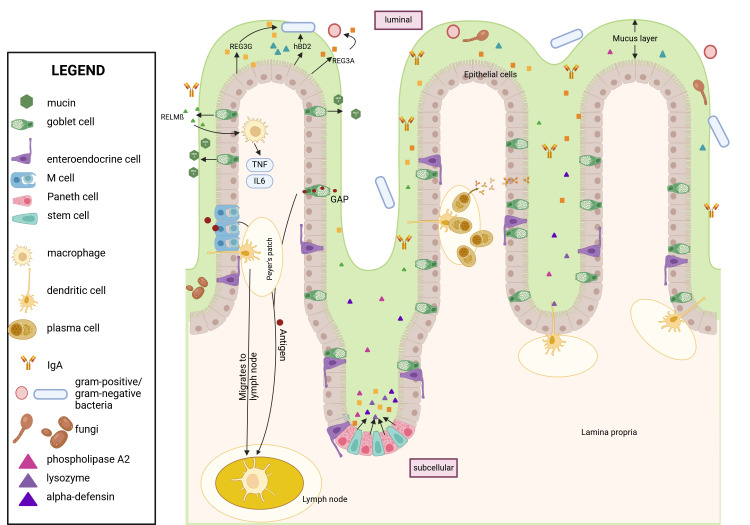
Structure of the intestinal wall during a physiologic/healthy state The intestinal epithelial layer is covered by a mucus layer consisting of mainly mucin-2 that separates the intestinal epithelial cells from the luminal content, including bacteria and antigens. M cells and GAPs transport antigens through the barrier to APCs such as macrophages and dendritic cells, which then translocate to Peyer’s patches. APCs present the antigen to T cells, which induce a B cell response. B cells migrate to the lamina propria as plasma cells and secrete IgA. IgA is transported via polymeric immunoglobulin receptor to the surface of epithelial cells and shed into the mucus to neutralize bacteria. Antimicrobial peptides such as REG3s, defensins, lysozyme, and RELM-beta are secreted into the mucus via epithelial, goblet, and Paneth cells, which reside at the crypts next to intestinal stem cells. These peptides kill bacteria directly and promote immune cells. RELM-beta activates macrophages to kill bacteria indirectly. Created with biorender.com. *Note:* APC, antigen-presenting cell; GAP, Goblet-cell antigen passage; hBD2, human beta defensin 2; IgA, immunoglobulin A; IL, interleukin; REG3, regenerating islet-derived-3; RELM-beta, resistin-like molecule beta; TNF, tumor necrosis factor.

The intestinal barrier separates the luminal from the subcellular compartment of the intestine. The luminal compartment refers to the inner cavity of the intestine where food passes through and bacteria reside, and the subcellular compartment includes structures below the intestinal epithelial cells, such as immune cells. The barrier consists of three functionally different parts: mucus, epithelial cells, and immune cells. The physical barrier formed by epithelial cells is the first obstacle for bacterial translocation (Figure 1).

The intestinal epithelial barrier comprises different cell types that differ in morphology and function. Epithelial cells derive from intestinal stem cells and differentiate into enterocytes, goblet cells, Paneth cells, intestinal microfold cells (M cells), and enteroendocrine cells (Figure 1). The main cell type is enterocytes, which absorb nutrients and secrete antimicrobial peptides (AMPs). Goblet cells secrete mucus, present luminal antigens to dendritic cells in the lamina propria, and secrete AMPs. Paneth cells are located in the crypts and produce AMPs. M cells transcytose antigens to present them to Peyer’s patches. Finally, enteroendocrine cells produce local hormones regulating satiety and hunger.

**Figure 2:**
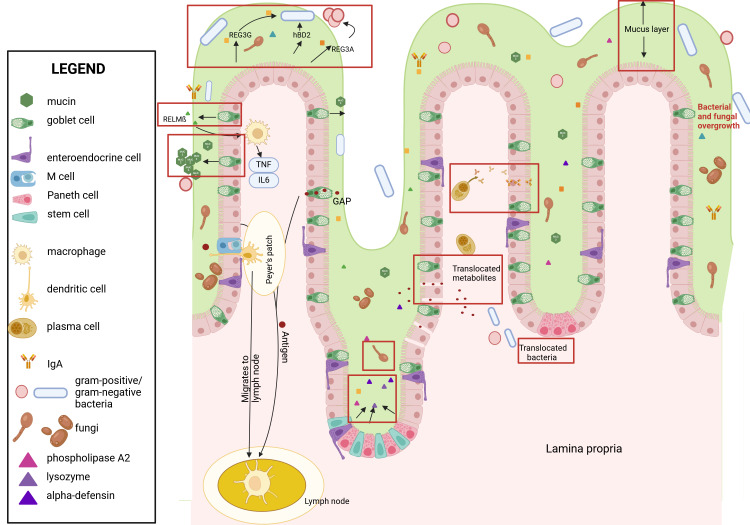
Ethanol's effects on the intestinal wall Ethanol leads to numerous changes in the intestinal immune response (see red boxes). These include a thicker mucus layer, intestinal dysbiosis, and fungal and bacterial overgrowth, resulting in longer contact time of bacteria with epithelial cells. Paracellular spaces enable translocation of bacterial metabolites. Viable bacteria translocate via mechanisms that require further investigation. Production and secretion of antimicrobial peptides and IgA are reduced, which decreases defense mechanism against pathogens. Immune cells such as M cells and dendritic cells are decreased while goblet cells are increased. Created with biorender.com. *Note:* GAP, goblet-cell antigen passage; hBD2, human beta defensin 2; IgA, immunoglobulin A; IL, interleukin; REG3, regenerating islet-derived-3; RELM-beta, resistin-like molecule beta; TNF, tumor necrosis factor.

Chronic alcohol consumption leads to disruption of the intestinal barrier, including a thicker mucus layer and widened paracellular spaces (spaces between epithelial cells), which enable translocation of MAMPs and eventually bacteria (Figure 2). 8,11,12 However, bacteria are larger than the disrupted paracellular spaces, indicating an alternative route for bacteria to cross the gut barrier.^8^

Increased thickness of the mucus layer reduces bacterial mobility, which results in an increased contact time between bacteria and epithelial cells, facilitating translocation.^13^ At the same time, various molecules present in the mucus layer that can provide an antimicrobial response in a healthy state—such as AMPs that directly kill bacteria as well as soluble immunoglobulin A (sIgA) molecules that can bind to bacteria—are decreased in patients with ALD and in mice fed a chronic ethanol diet.^14^–^17^ Hence, bacteria invading the mucus layer are less likely to be killed by AMPs or bound by sIgA. Patients with alcohol-associated hepatitis can be treated with steroids (e.g., prednisolone) depending on their clinical state. Treating dysbiosis and bacterial translocation with antibiotics seems to be a feasible addition to the treatment. When mice are treated with nonabsorbable antibiotics (i.e., antibiotics that stay in the gut and are not absorbed into the systemic circulation), gut dysbiosis and inflammation are reduced; however, treating patients with alcohol-associated hepatitis with antibiotics systemically (i.e., the antibiotics also enter the circulation) does not show a benefit over prednisolone therapy alone.^1^,^18^,^19^ Besides the difference of administering antibiotics that are absorbable vs. nonabsorbable, possible reasons for these different outcomes could include the presence of antibiotic-resistant bacteria, secondary effects of antibiotics, and effects of antibiotics on the immune system.

Alcohol compromises the enterocyte barrier by nitrooxidative stress and altered tight junctions.^20^ Moreover, increased apoptosis of enterocytes has been linked to ethanol-induced intestinal barrier dysfunction.^21^ Both of these effects impact the intestinal immune system. To understand the importance of the intestinal immune system in ALD, factors and components such as epithelial cells and their interactions must be highlighted.

The following sections discuss individual cell types found in the intestinal barrier and alcohol’s effect on these cells.

### Enterocytes

Enterocytes absorb nutrients in the small intestine as well as secrete and respond to cytokines. As part of the physical barrier, the enterocytes release AMPs such as beta-defensins, cathelicidins, and regenerating islet-derived-3 (REG3) lectins. AMP secretion is triggered by the recognition of bacterial molecules by Toll-like receptors (TLRs), such as TLR4, and nucleotide-binding oligomerization domain-containing proteins (NODs), such as NOD2, which are membrane-bound receptors on enterocytes.^22^, ^23^ Humans express REG3A—also known as HIP/PAP (hepatocarcinoma-intestine-pancreas/pancreatitis-associated protein)—and REG3G, whereas Reg3b is only present in mice. REG3A kills Gram-positive bacteria after binding membrane phospholipids and forming a membrane-permeabilizing pore.^24^ LPS, which is part of the outer membrane of Gram-negative bacteria, inhibits the REG3A-mediated pore formation; therefore, the REG3A ability to kill Gram-negative bacteria is limited.^24^ Human beta-defensins (hBD) consist of six isoforms, and some, such as hBD2, are induced by microbial products.^25^ hBD2 binds directly to the membrane of Gram-negative bacteria through LPS and induces pore complex formation.^25^ It can ultimately reduce inflammation by binding to host cell surface proteins on immune cells.^25^ Several AMPs, including REG3 and hBD2, are reduced in patients with ALD.^14^,^26^,^27^ Administration of hBD2 to mice fed a chronic ethanol diet (Lieber-DeCarli diet for 6 to 8 weeks) ameliorates liver damage by inducing interleukin-17A (Il17A) and Il22 and by increasing abundance of regulatory T (T_reg_) cells in the small intestine and the liver.^27^ Furthermore, hBD2 modulates the gut microbiota composition.^27^

Aryl hydrocarbon receptor (AhR) is a ligand-activated transcription factor that is inactive in the cytosol. Once activated by xenobiotics, dietary, microbial, or metabolic cues, such as the microbial metabolite indole, AhR relocates to the nucleus (Figure 1),^28^–^30^ where it induces transcription of targeted genes, such as zonulin-1 and occludin.^28^ Alcohol is associated with a lower production of intestinal AhR ligands by the gut microbiota, such as indoles, and with lower AhR signaling.^31^ The effect can be reversed when ethanol-fed mice (Lieber-DeCarli diet for 15 days) are treated with the microbial metabolite indole-3-acetic acid.^31^ Similarly, delivering AhR agonists via engineered bacteria protected mice against ethanol-induced liver disease by upregulating intestinal genes, including Il22, Reg3b, and Reg3g.^32^ In contrast, mice with an intestinal epithelial cell-specific deletion of AhR showed more severe liver disease after ethanol feeding than did control mice.^31^ However, patients with alcohol-associated hepatitis have a higher systemic AhR activity, which correlates with mortality. It is likely that the source for systemic AhR activity is not only microbiota-derived ligands, but also systemic AhR ligands such as bilirubin.^33^

Other functions of enterocytes include transepithelial transport of immunoglobulin A (IgA) and secretion of Ly6/Plaur domain-containing 8, a protein that binds to flagella of certain bacteria to create a spatial separation from the intestinal epithelial cells.^34^, ^35^

### Goblet Cells

A mucus layer covers epithelial cells throughout the whole intestine. The composition of the mucus layer differs in various parts of the intestine. In the colon, it is composed of two layers.^36^ The inner layer attached to the epithelial cells is devoid of bacteria, whereas the less dense outer layer is colonized by bacteria and can be easily washed off.^36^ There is only one layer of mucus in the small intestine, into which AMPs and IgA are secreted to reduce microbial adherence.^36^ Mucus consists predominately of O-glycosylated mucins, which are secreted by goblet cells. They can be secreted as gel-forming mucins or can be transmembrane-bound mucins forming the glycocalyx layer to protect against pathogens.^36^ The most abundant secreted mucin is mucin-2 (MUC2).^36^ MUC2 stays attached to goblet cells until cleaved by the protease meprin beta and released to the mucus layer.^37^, ^38^ Commensal bacteria can degrade mucin-glycans to extract energy and to feed themselves and their host.^37^

Chronic alcohol exposure increases Muc2 expression and induces thickening of the mucus layer in the ileum and colon of rats, as well as in the duodenum of humans.^12^, ^39^ Mice deficient in Muc2 show a thinner mucus layer and less severe ethanol-induced liver disease compared with wildtype controls.^12^ They develop a leakier gut barrier and show less translocation of LPS to the systemic circulation.^12^ The latter is explained by a reduction in bacterial overgrowth, a heightened intestinal immune surveillance and increased expression of the AMPs Reg3b and Reg3g.^12^ Chronic alcohol consumption (Lieber-DeCarli diet for 6 to 8 weeks) increases the number of goblet cells in humans and mice. 39–41 Administration of a synthetic retinoid reverses this effect in mice, resulting in less damage to the gut barrier and reduced ethanol-induced liver disease.^39^

One of the most unique features of goblet cells are goblet cell-associated antigen passages (GAPs), which allow luminal antigens to pass through the cells and be presented to lamina propria dendritic cells. This induces an adaptive and tolerogenic immune response (i.e., an immune response that tolerates beneficial bacteria and their antigens).^42^ GAPs are formed as a response to acetylcholine binding to the muscarinic acetylcholine receptor 4.^43^ In the healthy state, GAPs are open in the small intestine but closed in the colon.^44^ They open in case of disruption in microbial sensing (i.e., detection of microbial signals or antigens in the gut lumen).^44^ How alcohol changes the function of GAPs is unknown and requires future investigations.

Finally, goblet cells produce AMPs such as resistin-like molecule beta (RELM-beta) and trefoil factor, which protect epithelial cells against microbial invasion.^45^ RELM-beta promotes inflammation and is effective against helminth (parasitic worms) infection via mediating goblet cell–macrophage crosstalk.^46^–^48^

### Paneth Cells

Paneth cells are located at the base of the crypts next to intestinal stem cells. Their predominant function is to maintain intestinal barrier integrity by secreting AMPs (e.g., lysozyme, alpha-defensins, phospholipase A2) and REG3 lectins (e.g., REG3G) into the inner mucus layer.

REG3G is essential for separation between microbiota and intestinal epithelial cells.^23^, ^49^ Alcohol downregulates REG3G expression in duodenal biopsies from patients with alcohol use disorder as well as Reg3b and Reg3g expression in mice.^9^,^12^,^14^ Decreased Reg3g levels promote attachment of bacteria to epithelial cells and enhance bacterial translocation, which causes an inflammatory response in the liver and worsens ethanol-induced liver disease in mice.^14^ Overexpression of Reg3g in intestinal epithelial cells shows the opposite effect. It restricts bacterial colonization of mucosal surfaces, reduces bacterial translocation, and protects mice from ethanol-induced liver disease.^14^

Alpha-defensin accounts for about 70% of the Paneth cell’s antimicrobial activity against Gram-positive and Gram-negative bacteria, fungi, and viruses.^50^ Chronic ethanol feeding (Lieber-DeCarli diet for 12 weeks, 8 weeks, and 30 days) reduced Paneth cell alpha-defensin expression in the small intestine in mice and supplementation of human alpha-defensin ameliorated ethanol-induced liver disease in mice.^51^, ^52^

In summary, alcohol impairs the antimicrobial activity in Paneth cells and facilitates bacterial translocation.

### M Cells

Organized mucosal lymphoid tissues in the epithelium of the intestine, such as Peyer’s patches, isolated lymphoid follicles, and colonic patches, are surrounded by M cells (Figure 1). These cells endocytose and transcytose luminal antigens and deliver them to antigen-presenting cells, such as macrophages and dendritic cells, localized in specific lymphoid tissue in the lamina propria.^53^ The decision whether to induce an immune reaction or tolerance is determined by antigen presentation to T cells, which subsequently induce B cell differentiation into IgA-secreting plasma cells.^54^ Each M cell is accompanied by a B cell that is crucial for the development of M cells; however, the function of the B cell in the immune response is still elusive.^55^

M cells showed marked changes in the ultrastructure of the cell, including mitochondrial swelling with loss of matrix density, dilation of the endoplasmic reticulum, and cytoplasmic vacuolization, in rats that had been fed ethanol for 45 days.^56^ The total number of M cells and the absolute number of B cells and T cells in Peyer’s patches decreased after chronic ethanol consumption (Lieber-DeCarli diet for 5 weeks) in mice (Figure 2).^57^ Transcytosis of antigens by M cells is upregulated when challenged with bacteria.^58^

### Enteroendocrine Cells

Less than 1% of the intestinal mucosal cells are enteroendocrine cells. Various enteroendocrine peptides—such as somatostatin, motilin, cholecystokinin, neurotensin, vasoactive intestinal peptide, enteroglucagon, gastric inhibitory peptide, glucagon-like peptide 1 and 2, cleaved peptide YY, oxyntomodulin, and histamine—are stored inside these cells and released upon stimulation of G protein coupled receptors.^63^ Stimulation of the receptors is dependent on their location in the gut. Enteroendocrine cells in the proximal small intestine carry receptors for nutrients, whereas enteroendocrine cells in the distal small intestine release peptides after stimulation with microbial metabolites.^63^, ^64^

Chronic alcohol consumption affects the enteroendocrine system in the duodenal mucosa in humans and rats. In rats fed a chronic ethanol diet (ethanol concentration of 5%, which was increased weekly by 5% until the concentration reached 25% for 6 months), intestinal and plasma levels of somatostatin were reduced.^65^ In humans with chronic alcohol misuse, the number of enteroglucagon- and gastric inhibitory peptide-releasing cells slightly increased.^65^

### Intestinal Stem Cells

All intestinal epithelial cells are derived from intestinal stem cells located at the base of crypts. During the process of differentiation, cells move up to the villus before going into apoptosis after 5 days.^59^ Constant renewal of the epithelial cells secures a healthy intestinal barrier. Intestinal stem cells are upregulated during acute injury to promote epithelial regeneration. This process requires stimulation of pathways such as the gp130-YAP-Notch pathway and the Wnt/beta-Catenin pathway.^60^ Studies found conflicting effects of ethanol on intestinal stem cells. In one study, chronic ethanol consumption in mice (Lieber-DeCarli diet for 8 weeks) suppressed proliferation of intestinal stem cells via dysregulation of beta-catenin in mice and organoids.^61^ In contrast, another study showed enhanced proliferative activity and increased Wnt-target gene expression.^62^ Therefore, future studies investigating alcohol’s effects on the delicate balance between differentiation and proliferation of intestinal stem cells can move the field forward.

### Summary

The intestinal barrier comprises different cell types that help to maintain barrier function and protect against translocation of bacteria. Alcohol disrupts these mechanisms. Reduction of AMPs leads to impaired bacterial killing, dysbiosis, and a compromised gut barrier function.

## Intestinal Microbiome and Gut Dysbiosis

The gut microbiota is composed of the bacterial microbiome, virome (collection of all viruses), and mycobiome (collection of all fungi). Alterations in the gut bacterial microbiome, virome, and mycobiome are associated with the severity of ALD.^9^,^66^–^69^ Bacterial overgrowth combined with decreased diversity have been reported in mice with ethanol-induced liver disease and humans with ALD. An increased number of Proteobacteria, *Enterobacteriaceae*, and *Streptococcus* and a reduced number of *Bacteroides*, *Akkermansia*, and *Faecalibacterium* were identified in mice fed ethanol.^9^ Additionally, an increased number of *Candida albicans* was found in humans.^68^ Transplantation of feces from patients with alcohol-associated hepatitis into mice that are not colonized by any bacteria (i.e., germ-free) resulted in intestinal barrier disruption and liver injury.^6^ On the other hand, germ-free mice had more severe liver injury than conventional mice when challenged with a single binge of ethanol at a dose of 3 g/kg body weight, indicating a protective effect of some microbes.^70^

Recent studies found that specific bacteria contribute to ALD. For instance, cytolysin, an exotoxin produced by *Enterococcus faecalis*, aggravated ethanol-induced liver damage in mice.^71^ When cytolysin production was prevented by precisely killing cytolysin-producing *Enterococcus faecalis* strains with phage therapy, liver injury was reversed.^71^

In summary, alcohol alters the composition of the microbiome, which in turn shapes the intestinal immune system. Beneficial gut bacteria decrease, while harmful bacteria increase and contribute to ALD.

## Immune Cells in the Gut and Alterations Due to Alcohol

An important function of the immune system is to prevent infection while being tolerant to commensal bacteria and food antigens in the gut. Specialized cells, including epithelial cells and bone marrow-derived immune cells, are required to maintain this balance. Bone marrow-derived immune cells can be divided into monocytes and lymphocytes. Monocytes develop, for example, into dendritic cells or macrophages. Lymphocytes are part of the adaptive immune system and include T and B lymphocytes, innate lymphoid cells, and plasma cells. All these cells are present in the intestine.

### Dendritic Cells

The function of intestinal dendritic cells is to promote antigen tolerance, heighten immune surveillance, and activate the adaptive immune system.^72^ Dendritic cells can be found in Peyer’s patches, mesenteric lymph nodes, and the lamina propria.^76^ Two main types of these cells exist—conventional type 1 dendritic cells (cDC1) and conventional type 2 dendritic cells (cDC2)—and are distinguished by the presence or absence of two surface proteins called cluster of differentiation (CD) proteins CD103 and CD11b. cDC1 cells, which carry CD103 but lack CD11b (CD103+ CD11b−), are mostly present in the colonic lamina propria.^72^ cDC2 cells carry CD11b but may or may not carry CD103 (CD103+ CD11b+ and CD103− CD11b+); they are most abundant in the small intestinal lamina propria.^72^

**Figure 3:**
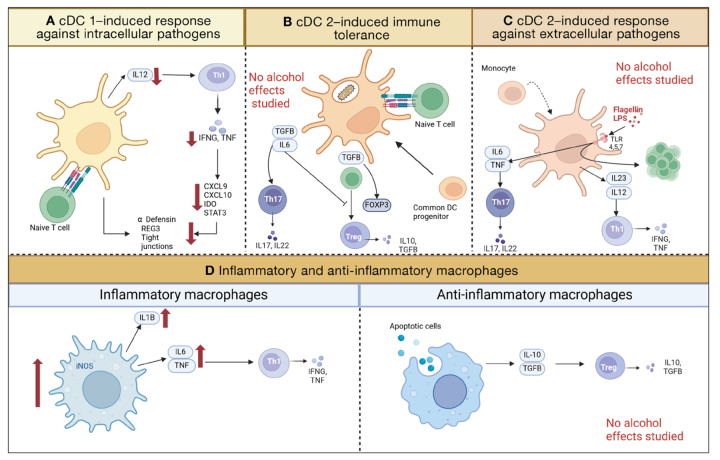
Functions of monocyte-derived cells in the intestine, including three subsets of conventional dendritic cells (cDC) and macrophages Red arrows indicate decreases or increases due to ethanol. (A) cDC1 (CD103+ CD11c−) prime naïve T cells to become Th1 cells by secreting IL12. Th1 secrete INFG and TNF, which induce immune response via various cytokines. The dendritic cells and these cytokines are part of the regulation of antimicrobial peptides and tight junction proteins. Ethanol diminishes the number of cDC1, including their downstream effects. (B) cDC2 (CD103+, CD11+) prime naïve T cells to become Th17 or T_reg_ cells, depending on the cytokines they secrete after phagocytosing bacteria. (C) cDC2 (CD103− CD11c+) respond to microbial metabolites binding to TLRs and induce Th17, Th1, and T cell proliferation. (D) Macrophages can exhibit an inflammatory or anti-inflammatory state. Inflammatory macrophages induce Th1 differentiation and are increased in ethanol-induced liver disease. Anti-inflammatory macrophages induce T_reg_ cell differentiation. Created with biorender.com. *Note:* cDC, conventional dendritic cells; CXCL, chemokine (CXC-motif) ligand; FOXP3, Forkhead-Box-Protein P3; IDO, indoleaminepyrrole 2,3-dioxygenase; IFNG, interferon gamma; IL, interleukin; iNOS, nitric oxide synthase; LPS, lipopolysaccharide; REG3, regenerating islet-derived-3; STAT3, signal transducer and activator of transcription 3; TGFB, transforming growth factor beta; Th cells, T helper cells; TLR, Toll-like receptor; TNF, tumor necrosis factor.

Each subset of dendritic cells exhibits several specialized functions. cDC1 and CD103+ cDC2 directly capture bacteria when these are translocated through M cells or GAPs. These cells process the bacteria and present antigens to T cells in mesenteric lymph nodes.^72^ Both cDC1 and CD103+ cDC2 also secrete IL12 and promote differentiation to T_reg_ cells (Figure 3).^73^

cDC1 cells primarily act to induce a response against extracellular pathogens. IL12 secretion by cDC1 leads to the differentiation of T cells into interferon-gamma (IFNG)-secreting Th1 and CD8+ effector T cells.^74^, ^75^ IFNG plays a pivotal role in immune protection against intestinal pathogens by stimulating the induction of AMPs (Figure 3A).^73^,^76^–^78^ Hence, cDC1 are important to establish an adaptive immune response while promoting tolerance to antigens. A study investigating the effect of chronic ethanol feeding (Lieber-DeCarli diet for 8 weeks, NIAAA model) on cDC1 in mice found that whereas the overall number of dendritic cells in Peyer patches and the small and large intestinal lamina propria was increased, the absolute number of cDC1 was decreased, resulting in lower IL22 levels.^79^, ^80^ This resulted in fewer Th1 and CD8+ T cells secreting IFNG and was associated with a lower adaptive immune response.^79^, ^80^ Consistent with these data, chronic ethanol exposure (Lieber-DeCarli diet for 8 weeks) has been shown to lead to a downregulation of IFNG in mice and a decrease of Reg3s and alpha-defensins.^78^ These changes affected the intestinal microbiota signature, promoted a reduction of *Akkermansia muciniphila*, and resulted in disruption of intestinal tight junctions.^78^ Lack of cDC1 also aggravated ethanol-induced tight junction disruption and promoted bacterial translocation.^79^

Among the cDC2 cells, the CD103+ subset serves to induce immune tolerance, whereas the CD103− subset helps to induce responses against extracellular pathogens. The CD103+ CD11b+ cDC2 subset induces IgA, directs gut-specific lymphocytes to the gut (lymphocyte-homing), and regulates the differentiation and expansion of CD4+ T cells into T_reg_ cells (Figure 3B). In a steady state, CD103+ CD11b+ cDC2 express av-beta-integrin, which activates latent transforming growth factor B (TGFB) and the transcription factor FOXP3, resulting in T_reg_ cell differentiation.^73^, ^81^ During inflammation, the CD103+ CD11b+ cDC2 cells secrete IL6, which together with TGFB induces T helper 17 (Th17; a specialized set of T helper cells that secrete IL17) cell differentiation and inhibits FOXP3, thereby preventing autoimmunity (Figure 3B).^73^, ^82^

CD103− CD11b+ cDC2 produce IL22 and induce a Th17 cell response after stimulation with the TLR5 agonist flagellin (Figure 3C).^83^

The effect of alcohol on dendritic cells other than cDC1 is elusive. In the systemic circulation, alcohol has been shown to modify function and cytokine production of human monocyte-derived dendritic cells, but further studies of intestinal dendritic cells may provide further insight.^84^

### Macrophages

The intestinal lamina propria is the largest reservoir of macrophages in the body.^85^ Even though there are macrophages in the muscularis propria (a layer of smooth muscle in various organs), these have not been studied in the setting of ALD.^86^ Macrophages can be derived from either bone marrow cells or local stem cells.^86^ TGFB and IL8, produced by epithelial cells and mast cells, are stored and released in the lamina propria extracellular matrix (stroma). These cytokines attract monocytes in the systemic circulation, prompting them to differentiate into resident macrophages.^87^ They are unevenly distributed within the gastrointestinal tract, with the highest numbers found in the colon.

Intestinal macrophages can be subdivided depending on their expression of surface markers, activation pathways, and immune responses (Figure 3D). Monocyte-derived mature macrophages produce anti-inflammatory cytokines such as IL10, show an enhanced phagocytic activity, acquire scavenger-receptor, and stay nonresponsive to TLR ligation.^88^ They can be repolarized to monocyte-derived inflammatory macrophages expressing tumor necrosis factor (TNF), IL1-beta (IL1B), and IL6.^89^ A third population of self-maintaining macrophages, derived from embryonic precursors and adult bone-marrow–derived monocytes, express higher levels of genes involved in development and tissue support (e.g., angiogenesis and epithelial cell differentiation).^90^

In a healthy gut, intestinal macrophages are highly phagocytic. They release fewer cytokines in response to LPS compared to other macrophages because they lack CD14, an important co-receptor for the LPS response.^91^ Instead, intestinal macrophages secrete anti-inflammatory cytokines such as IL10, which, together with TGFB, promotes T_reg_ cell differentiation (Figure 3).^91^

Like dendritic cells, macrophages help maintain a balance between immune tolerance and activation. Intestinal macrophages phagocytose apoptotic epithelial cells and modulate the luminal microbiota during health.^86^ They protect epithelial cells from toxic metabolites by testing the absorbed fluids and halting absorption if fungal products are detected.^86^, ^92^

Intestinal barrier disruption and microbial dysbiosis increase antigen presentation to lamina propria macrophages. CD68/TNF-positive monocytes and macrophages are increased in the duodenal lamina propria of patients with ALD and the jejunal lamina propria in ethanol-fed mice while the overall population of macrophages is unaltered.^1^ This effect can be blunted when treated with nonabsorbable antibiotics, indicating that bacterial overgrowth and dysbiosis lead to the activation of lamina propria macrophages.^1^ Conversely, the number of macrophages is reduced in the lamina propria of the proximal colon in mice fed ethanol.^93^, ^94^

In mice, intestinal macrophages express a higher level of inflammatory cytokines such as lipocalin, TNF, Il1B, and colony-stimulating factor 1 after chronic and binge ethanol feeding with or without LPS. Colony-stimulating factor 1 is important for maintaining the intestinal macrophage population.^90^ Macrophages can be classified by the expression of surface markers into more inflammatory (often termed M1) macrophages expressing nitric oxide synthase (iNOS) and anti-inflammatory (often termed as M2) macrophages expressing arginase1 (ARG1). *Arg1* messenger RNA (mRNA) expression, but not *iNos* mRNA expression, is reduced in the proximal colon of mice fed ethanol for 16 days. This effect can be reversed by treatment with a butyrate derivative.^94^ This indicates that ethanol consumption stimulates inflammatory macrophages that phagocytose microbes to produce inflammatory cytokines and initiate an immune response.^94^

In summary, alcohol leads to an increase of inflammatory macrophages in the jejunum and to a decrease of macrophages in the colon. Current data do not specify the effect of alcohol on specific subsets of macrophages in the intestinal lamina propria.

### Lymphocytes

#### CD8+ resident memory T cells

Resident memory T cells (TRMs) are a specific subset of T cells present in the mucosa of the intestine. The CD8+ T-cell pool in the intestinal mucosa is principally composed of TRMs. Their key tasks are immunosurveillance and host defense. TRMs initiate a fast and targeted immune response in mucosal tissues after antigen binding, without being stimulated by antigen-presenting cells.^95^ After antigen binding, they secrete inflammatory cytokines and chemokines, including IFNG, TNF, granzyme B, and perforin.^95^

Recently, a study investigated the effect of alcohol on duodenal TRMs. Patients with ALD show an increased proportion of late apoptotic TRMs and a reduction of viable TRMs in the duodenum.^96^ The reduced number of TRMs was associated with a disturbed immunosurveillance and was inversely correlated with markers of bacterial translocation.^96^, ^97^

In summary, alcohol leads to apoptosis of TRMs in the duodenum and disrupted immune vigilance. Alcohol’s effects on TRMs in other parts of the intestine require further studies.

#### Regulatory B cells

Regulatory B cells, also called B10 cells, are characterized by the presence of CD1d and produce IL10.^98^ They are induced in a chronic inflammatory environment in gut-associated lymphoid tissues, such as Peyer’s patches.^98^ B10 cells suppress the progression of intestinal inflammation by downregulating inflammatory cascades.^98^ Further anti-inflammatory effects include promotion of T_reg_ cells and inhibition of Th1 and Th17 cell differentiation.^99^ Gut bacteria can indirectly promote B10 development through induction of IL6 and IL1B.^99^ Hence, commensal bacteria promote B10 development independent of TLR activation to boost immune tolerance.^99^ Adoptive transfer of regulatory B cells reduces inflammation in an Il10-dependent manner in mice lacking B cells and subjected to dextran sulfate sodium-induced intestinal injury.^100^ The effect of alcohol on regulatory B cells in the intestine is not known.

#### Innate lymphoid cells

Innate lymphoid cells (ILCs) reside mostly in the tissue throughout the body and do not express surface proteins of B- and T-cell lines.^101^ Various types of ILCs are present in the intestinal mucosa and lamina propria, functioning analogously to polarized CD4+ T-cell subsets.

Group 1 ILCs (ILC1s), including natural killer cells and noncytotoxic ILC1s, represent the counterpart to Th1 cells.^102^ They release IFNG at mucosal inflammation sites to fight intracellular pathogens such as viruses and bacteria.^103^ Group 2 ILCs (ILC2s) respond to parasite infection by secreting Th2-cell cytokines such as IL5, IL9, and IL13, and produce antimicrobial peptides to promote tissue repair.^103^–^105^

Group 3 ILCs (ILC3s) are analogous to Th17 cells, producing interleukins IL17 and IL22 to combat extracellular microbes such as bacteria and fungi.^102^ IL22 is linked to a regenerative response, including maintenance of epithelial barrier integrity as well as increased REG3G and REG3B expression.^106^–^108^ IL17 upregulates neutrophil chemokine production as part of an inflammatory response.^102^ Microbiota and microbial metabolites or stimulation via short-chain fatty acid receptors activates ILC3s directly or indirectly.^109^, ^110^ ILC3s co-localize with T cells in mesenteric lymph nodes, promote microbiota-specific RAR-related orphan receptor gamma-t positive (RORγt+) T_reg_ cells, and prevent their expansion as inflammatory Th17 cells.^111^ Overall, ILC3s orchestrate the immune response by inducing anti-inflammatory cells such as T_reg_ cells and restraining inflammatory cells such as Th17 cells.^111^, ^112^

In general, the proportion of ILCs is reduced in mice fed an ethanol diet (NIAAA model).^10^ ILC3s produce lower levels of Il22 as a result of ethanol-induced dysbiosis and lower intestinal levels of indole-3-acetic acid following chronic ethanol feeding in mice.^106^, ^113^ Administration of Il22 reverses ethanol-induced reduction of Reg3g and alpha-defensin.^106^ Engineered bacteria producing Il22 reduced ethanol-induced liver damage, inflammation, and bacterial translocation in mice.^113^ Furthermore, a PEGylated Tlr7 ligand upregulates intestinal expression of Il22, Reg3b and Reg3g, while Il22 deficiency abolishes the protective effect of the ligand in mice.^114^ Il22 expression is dependent on bacteria such as *Lactobacillus rhamnosus*, indicating that the microbiome shapes ILCs.^115^

The transcription factor AhR is not only expressed in intestinal epithelial cells, but also in ILCs. It regulates the maturation of ILC3s and secretion of IL22.^116^ Direct stimulation of AhR using indole-3-acetic acid or AhR agonists produced by engineered bacteria improves ethanol-induced liver disease in mice. This improvement is marked by increased expression of Il22 in ILC3 cells, as well as Reg3b and Reg3g, and reduced bacterial translocation to the liver.^32^, ^117^

Similar to T_reg_ cells, regulatory ILCs (ILC_regs_) produce IL10 to inhibit the innate immune response against intestinal inflammation.^118^ However, recent data show that IL10 is mainly produced by a subset of ILC2.^119^

Overall, ILCs play an important role in the intestinal immune system. Consumption of alcohol reduces and functionally impairs ILC3s. However, the effects of alcohol on ILC1 and ILC2 are still unclear.

#### IgA-secreting cells

All mucosal tissues in the human body are covered by IgA. IgA binds to both commensal and pathogenic bacteria to prevent them from crossing the epithelial barrier. In addition to its direct effects, sIgA neutralizes bacterial toxins and protects epithelial cells from the toxic stimulation.^15^ Two subtypes of IgA are present in the human intestine, IgA1 and IgA2, whereas mice express only one type of IgA.^16^, ^120^ IgA1 and IgA2 are important to maintain homeostasis of commensal bacteria and fight pathogens.^16^ More than 80% of all human plasma cells reside in the lamina propria and secrete IgA.^16^ IgA is transported from the lamina propria to the intestinal lumen through transcytosis, mediated by the polymeric immunoglobulin receptor (pIgR).^16^, ^121^ Endopeptidases cleave the luminal domain of pIgR, releasing the secretory component together with IgA to protect IgA from degradation.^121^, ^122^ Hence, pIgR regulates the amount of sIgA that coats pathogenic and commensal bacteria.^17^

IgA can be secreted via T cell-dependent and T cell-independent responses, depending on the type of bacteria. Commensal bacteria in the duodenum trigger T cell-independent responses originating from B cells that separate bacteria from intestinal epithelium and prevent T-cell activation.^123^ IgA binding to pathogenic bacteria is induced via the T cell-dependent pathway, leading to a reduction of their motility, growth inhibition, and greater distance to the intestinal surface.^124^, ^125^

Mice fed a chronic ethanol diet (NIAAA model) showed an increased number of antigen-specific IgA-secreting cells in the serum and in the liver.^126^ IgA was increased in the serum in patients with ALD, and patients with significantly elevated IgA levels showed more advanced disease than patients with normal IgA levels.^127^ PIgR is also expressed in the liver, specifically in cholangiocytes under healthy conditions.^17^ Patients with alcohol-associated hepatitis demonstrated an increased colocalization of pIgR and IgA in hepatocytes.^17^ pIgR-deficient mice developed increased liver injury, steatosis, and inflammation driven by increased bacterial translocation.^17^ Increased susceptibility to ethanol-induced liver disease was ameliorated by reexpressing pIgR in hepatocytes of pIgR-deficient mice, indicating that hepatic secretion of IgA into the bile is important to prevent bacterial translocation and reduce ethanol-induced liver disease.^17^ The number of IgA-secreting plasma cells in the lamina propria, fecal IgA level, and gene expression of pIgR were diminished in mice fed a chronic ethanol diet (NIAAA model and Lieber-DeCarli diet for 4 weeks).^126^,^128^,^129^ Interestingly, transfer of IgA-coated bacteria from ethanol-fed mice into wildtype mice impaired the lung immune system.^129^ Studies in patients with ALD are rare and demonstrate inconsistent results but show mainly slightly decreased sIgA with similar amounts of IgA-secreting cells in the intestine.^130^–^132^ Mice lacking IgA that were fed a chronic ethanol diet (NIAAA model and Lieber-DeCarli diet for 4 weeks), showed no aggravation of ethanol-induced liver disease, probably because of the compensatory roles of intestinal IgM and AMP.^128^ In summary, alcohol-induced deficiencies in IgA defenses can enhance ALD due to impaired antimicrobial defense.^113^

#### Mucosa-associated invariant T cells

Mucosa-associated invariant T cells (MAITs; Th1/Th17 phenotype) are present in the intestine, peripheral blood, and liver and play an important role in protecting against bacterial infections. They can be activated by microorganism-derived metabolites in a T cell receptor-dependent manner or by cytokines, including IL12, IL15, IL18, and type I interferon, in a T cell receptor-independent manner.^133^–^135^ Once activated, they secrete IFNG, TNF, granzyme, and IL17 to destroy bacteria-infected cells.^136^

Fewer but hyperactivated MAITs with impaired antibacterial and cytotoxic responses have been described in the blood and liver of patients with ALD.^137^, ^138^ The frequency of MAITs decreased with the severity of ALD, while the frequency of pyroptotic MAITs increased, indicating that activated MAITs are depleted by pyroptosis.^138^ Pyroptosis describes a form of cell death in which cells secrete proinflammatory cytokines. Pyroptotic MAITs in the systemic circulation correlate with the level of MAIT activation, intestinal enterocyte damage, soluble CD14, LPS-binding protein, and microbial translocation in patients with ALD or alcohol-associated liver cirrhosis.^138^ Pyroptosis was aggravated when stimulated with *Escherichia coli* or direct bilirubin.^138^ Blocking IL18 signaling reduced activation and frequencies of MAITs.^138^ Depletion of MAITs was not directly mediated by ethanol but rather an effect of long-term exposure to cytokines and bacterial antigens.^137^ Mice subjected to chronic ethanol feeding (NIAAA model) showed a significant overall reduction of MAITs in the lungs, liver, and intestine. However, activated MAIT increased in the intestine despite expressing fewer cytotoxic peptides such as IFNG and TNF.^139^

#### Th17 cells

Th17 cells have important barrier protective functions by regulating the immune response against extracellular bacteria and fungi; however, chronic activation of these cells might contribute to autoimmune disorders and chronic inflammation. They express cytokines such as IL17, IL22, IL6, and TNF.^140^

ROR-gamma-t is the master transcription factor for Th17 cells and participates in Th17 cell differentiation and development.^140^ Stimulation with TGFB and IL6 induces the expression of IL17 and IL23R in Th17 cells, while IL23 expands and maintains the Th17 population.^140^ Th17 cells and ROR-gamma-t–expressing T cells increase in the intestine in mice fed an ethanol diet (NIAAA model) due to upregulation of sphingosine kinase 1 activity and ROR-gamma-t activation.^10^ Treatment with 5-aminosalicylic acid decreases ethanol-induced liver injury and reverses gut inflammation by suppressing Th17 cells, indicating that Th17 cells are required for ethanol-associated gut inflammation.^10^

Elevated plasma IL17 levels are associated with an increase in peripheral and hepatic Th17 cells in patients with alcohol-associated hepatitis.^141^, ^142^ Th17 cells are antigen-specific, and intestinal *Candida albicans* induces Th17 cells in humans.^143^ Moreover, *Candida albicans* can migrate to different organs throughout the body.^144^ Patients with ALD show an overgrowth of *Candida albicans* in the intestine, leading to increased *Candida albicans-*specific Th17 cells in the liver.^68^, ^145^ Chronic ethanol administration in mice (NIAAA model and Lieber-DeCarli diet for 8 weeks) increases *Candida albicans*-specific Th17 cells, and transferring primed Th17 cells into mice fed a chronic ethanol diet (NIAAA model) worsens ethanol-induced liver injury.^145^ *Candida albicans*-primed Th17 cells are being reactivated by fungal antigens in the liver and contribute to the progression of ALD.^145^

## Clinical Implications

Changes in the intestinal barrier perpetuate progression of ALD via the described mechanisms. In cirrhosis, the advanced form of chronic liver disease, dysbiosis characterized by reduced diversity and small intestinal bacterial overgrowth, occurs largely independently of the underlying etiology.^146^, ^147^ Paracellular trafficking of bacterial metabolites as well as translocation of viable bacteria induce TNF overexpression in the small intestine via activation of TLR4.^148^–^151^ Furthermore, bacterial translocation to mesenteric lymph nodes leads to expansion of T cells and monocytes as well as production of IFNG and TNF.^152^ These primed immune cells spread through the circulation to different organs. TNF production in mesenteric lymph nodes is even more increased in cirrhotic patients with ascites (a condition characterized by buildup of fluid in the abdomen), while dendritic cells show an exhausted phenotype (i.e., lowered TNF-alpha production and relatively deficient phagocytosis and migration capacities). 153, 154 Bacteria also translocate directly to the liver and other compartments, and can cause infections such as spontaneous bacterial peritonitis.^148^,^149^,^155^,^156^ Due to clearance deficiency in patients with cirrhosis, bacteria and MAMPs reach the systemic circulation, initiating a systemic inflammatory response, a process called cirrhosis-associated immune dysfunction.^148^,^154^,^156^,^157^

All of these reactions—from intestinal barrier disruption and alterations in the intestinal immune system, particularly the activation of T cells that produce IFNG and TNF, to dendritic cells showing an exhausted phenotype—exacerbate cirrhosis. Consequently, treatment options for cirrhosis could involve restoring the intestinal barrier and modulating the intestinal immune system.

Besides the liver, alcohol-associated alterations in the intestinal immune system and dysbiosis also affect other organs, such as the lungs. Mice transplanted with fecal microbiota from patients with alcohol use disorder showed a higher susceptibility to pneumonia induced by *Klebsiella pneumoniae* or *Streptococcus pneumoniae.*^158^, ^159^ Ethanol-associated dysbiosis also led to a higher rate of *Klebsiella pneumoniae* infection independent of ethanol consumption in mice. 158, 159 Following *Klebsiella* infection, inflammatory cytokines in the lung increased, and the number of immune cells in the lung decreased.^159^ However, the number of immune cells in the intestine increased, indicating that intestinal T cell sequestration or dysregulated immune cell trafficking may impair pulmonary host defense.^159^ Mechanistic studies indicated that migration of immune cells from the intestine to the lung is partly driven by AhR.^159^, ^160^ Administration of a probiotic cocktail (containing *Bifidobacterium bifidum, Bifidobacterium lactis, Lactobacillus plantarum, Lactobacillus reuteri*, and *Lactobacillus rhamnosus)* or the microbial metabolite indole reduced the risk for alcohol-associated pneumonia.^160^

Alcohol-related dysbiosis affects alcohol-associated pneumonia by disrupting the intestinal immune system and pulmonary immune cell trafficking. Alcohol consumption increases the risk of pneumonia by 8% for each 10 to 20 g of alcohol consumed per day, due to impaired mucus-facilitated clearance, macrophage phagocytosis, recruitment of neutrophils, and reduction of peripheral natural killer cells; this alcohol consumption is associated with poorer outcomes.^129^,^159^,^161^,^162^

Generally, alcohol consumption leads to systemic immunosuppression and is often accompanied by comorbidities, partly explaining the worse outcomes.^162^ However, the studies mentioned above demonstrate that alcohol-associated dysbiosis is an important mechanism for promoting pneumonia independent of alcohol consumption. Therefore, pneumonia treatment in patients with alcohol use disorder could benefit from the addition of probiotics or indoles.

## Conclusions and Further Directions

The intestinal immune system has a variety of unique features to orchestrate the dichotomous functions of tolerance and initiation of an immune response. Alcohol has inflammatory effects on the intestine by a variety of mechanisms and disrupts the intestinal barrier directly and indirectly. A thicker mucus layer and reduced AMP levels impair bacterial mobility, leading to longer contact times of bacteria with epithelial cells, which promotes bacterial translocation.

Chronic administration of ethanol reduces the adaptive immune response via decreased numbers of cDC1 and IL12 and reduces immunosurveillance via decreased numbers of TRMs. Furthermore, ethanol causes a stimulation of inflammatory macrophages and an increase of *Candida albicans*-specific Th17 cells. ILC3s secrete IL22, which seems to be crucial for host defense as the administration of IL22producing bacteria or IL22 itself reduces ethanol-induced liver disease in mice.

Alcohol’s effects on some cells of the intestinal barrier, however, are still unclear and remain to be investigated, particularly alcohol’s impact on GAPs as well as on the differentiation and proliferation of intestinal stem cells. The influence of specific bacterial and fungal strains on the intestinal immune system and ALD also warrants further investigation. Additionally, alcohol’s effects on specific immune cells—including cDC2 subsets, TRMs outside the duodenum, B10 cells, ILC1, ILC2, and MAITs in the intestine—are still elusive. These topics warrant further exploration before attempting to reshape the intestinal immune system in patients with ALD using molecular targets. Additionally, immune cells such as Th17 have inflammatory effects while secreting cytokines to prevent bacterial overgrowth and regulating the intestinal barrier. Therefore, targeting these cells may even worsen ALD. Suppression of the inflammation using anti-inflammatory drugs such as 5-aminosalicylic acid seems to be a promising approach. Overall, targeting the immune system might be a therapeutic option to improve liver disease.

**KEY TAKEAWAYS**
- Chronic alcohol consumption disrupts the intestinal barrier, causing dysbiosis, increased mucus thickness, and reduced antimicrobial peptides. This promotes bacterial translocation and systemic inflammation, which contribute to alcohol-associated liver disease (ALD).
- Alcohol alters the intestinal immune system by disrupting the balance between immune tolerance and activation. This includes reduced regulatory T cells, increased pro-inflammatory responses (e.g., T helper 17 response and tumor necrosis factor production), and impaired antimicrobial defenses. These changes exacerbate gut inflammation, microbial translocation, and systemic immune activation, which aggravate liver damage in ALD.
- Modulating the intestinal immune system and restoring gut barrier integrity may offer potential treatments for ALD. Targeting gut inflammation with probiotics, antimicrobial peptides, or immune-modulatory strategies could mitigate liver injury.
